# Mapping and Ablation of Isolated Frequent Symptomatic Premature Atrial Contractions in Patients With Structurally Normal Heart

**DOI:** 10.3389/fcvm.2022.862659

**Published:** 2022-04-12

**Authors:** Bo He, Yi Li, Weiping Huang, Wenxi Yu, Fang Zhao, Xiaoyan Wu, Shuyuan Yao, Sunny S. Po, Zhibing Lu

**Affiliations:** ^1^Department of Cardiology, Zhongnan Hospital of Wuhan University, Wuhan, China; ^2^Cardiovascular Institute, Zhongnan Hospital of Wuhan University, Wuhan, China; ^3^Institute of Myocardial Injury and Repair, Wuhan University, Wuhan, China; ^4^Heart Rhythm Institute, Department of Medicine, University of Oklahoma Health Sciences Center, Oklahoma City, OK, United States

**Keywords:** premature atrial contraction, radiofrequency catheter ablation, right atrium catheter, isolated, structurally normal heart

## Abstract

**Background:**

The present study investigated the safety and efficacy of mapping and ablating isolated premature atrial contractions (PACs) in patients with a structurally normal heart, as well as whether the elimination of PACs by radiofrequency catheter ablation (RFCA) improved symptoms and the quality of life.

**Methods:**

Forty-three consecutive patients with frequent, symptomatic, and drug-refractory PACs, but without atrial tachyarrhythmias (≥5 beats), were enrolled. In all patients, we performed physical, laboratory, and imaging examinations to exclude structural heart disease. The quality of life questionnaire SF-36 before and 3 months after RFCA was performed in each patient.

**Results:**

Twenty-three men and 20 women with an average age of 52.6 ± 17.6 years were finally enrolled. The mean number of PACs was 21,685 ± 9,596 per 24 h, and the mean PACs' burden was 28.9 ± 13.7%. Short runs of tachycardia (<5 atrial beats) were observed in 32 patients (74.4%). All patients underwent successful RFCA without complications. The activation time at the successful ablation sites preceded the onset of the P-wave by 36 ± 7.6 ms. During 15 ± 8 months of follow-up, the recurrence of PACs was observed in 2 patients. The 24-h PAC burden was significantly reduced 3 months after RFCA (mean 0.5%, *p* < 0.05). The quality of life scores were significantly increased 3 months after RFCA (all *p* < 0.05).

**Conclusions:**

RFCA was feasible, safe, and effective to eliminate isolated frequent, symptomatic, and drug-refractory PACs in patients with a structurally normal heart. The elimination of PACs by RFCA significantly improved symptoms and the quality of life.

## Introduction

Radiofrequency catheter ablation (RFCA) has been the first-line therapy for a variety of tachyarrhythmias, including paroxysmal supraventricular tachycardia, atrial tachycardia, atrial flutter, and symptomatic atrial fibrillation (1–3). Frequent premature atrial contractions (PACs) in patients without structural heart disease are usually symptomatic and refractory to antiarrhythmic drugs (AADs) (4–6). Without proper treatment, frequent PACs could induce PAC-related cardiomyopathy (7–9). To date, there are no guidelines or expert consensus providing recommendations on RFCA for PACs. Recently, a few reports have demonstrated the feasibility and efficacy of RFCA for symptomatic PACs related to atrial fibrillation (4, 5, 10). However, the mapping and ablation of PACs are usually challenging as catheter manipulation in the atrium often provokes PACs, making it hard to differentiate these PACs from the clinical PACs due to the small amplitude of P wave.

In the present study, we investigated the feasibility, safety, and efficacy of mapping and ablating isolated PACs in patients with a structurally normal heart, as well as whether the elimination of PACs by RFCA improved symptoms and the quality of life.

## Methods

### Study Population

Consecutive patients presented with symptomatic PACs between January 2019 and June 2021 at Zhongnan Hospital of Wuhan University were enrolled. Physical examination, chest x-ray, surface electrocardiogram (ECG), 24-h Holter monitoring, and 2-D Doppler echocardiogram were performed to exclude structural heart diseases. Patients with atrial tachycardia (≥5 atrial beats), atrial flutter, or atrial fibrillation were excluded in the present study. The indications for RFCA include one of the following criteria: (1) cardiomyopathy suspected to be caused by frequent PACs; (2) AADs are ineffective, not tolerated, or not preferred for long-term therapy; (3) the number of PACs/24 h >10,000. The study protocol was approved by the ethics committee of Zhongnan Hospital of Wuhan University. We conducted it in accordance with the Declaration of Helsinki and the International Conference on harmonization Guidelines for Good Clinical Practice. Written informed consent was obtained from all participants.

### Electrophysiological Study

An electrophysiological study was performed in the fasting state after discontinuance of AADs for at least five half-lives. Under local anesthesia with 2% lidocaine, a decapolar catheter was positioned *via* the left subclavian vein or the right femoral vein into the coronary sinus (CS). Simultaneously, a quadripolar catheter was positioned *via* the right femoral vein into the high right atrium (HRA) or right atrial appendage (RAA) for recording and mapping.

Electrophysiological mapping was performed in all patients with spontaneous PACs or PACs induced by isoproterenol infusion (2–20 μg/min). Surface ECG and intracardiac bipolar electrograms were simultaneously recorded by the electrophysiological laboratory system (Lead 7000, Jinjiang Inc., Sichuan province, China). The P-wave morphology of ectopic PACs on surface ECG and the activation sequence of CS and HRA were used to approximate the origin site of the PACs. A positive ectopic *P* wave on lead V1, a distal-to-proximal CS activation, and a later HRA activation than CS indicate a left-side origin. Contrarily, a negative ectopic P wave on lead V1, a proximal-to-distal CS activation, and an earlier HRA activation than CS indicate a right-side origin.

**Figure 1:**
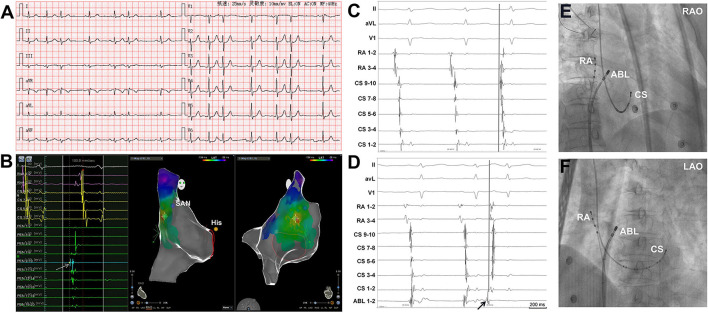
Simultaneous recordings of the right atrium (RA) and coronary sinus (CS) showing the differentiation of spontaneous PACs from PACs induced by catheter manipulation. **(A)** Surface ECG tracings during spontaneous PACs showing the P-wave morphology. The P-wave morphology in most of the leads is not clearly seen. **(B)** The 3D mapping shows that the origin site of the PACs is located at the high crista terminalis. The light blue dot indicates the earliest activation point of PACs. The dark blue dot indicates the sinoatrial node. The yellow dot indicates the His bundle. A double sharp atrial potential was recorded in the PentaRay catheter 9-10 (PEN 9-10, the arrow and the light blue dot) and the first atrial potential precedes surface ECG P wave in lead V_1_ by 40 ms. The RVA channels were connected to the RA catheter. **(C)** Surface ECG, RA, and CS tracings during spontaneous PAC and PAC induced by mapping catheter manipulation **(D)**. During spontaneous PAC, the proximal RA activation is earlier than the distal RA activation and the atrial activation sequence in CS is from proximal to distal. The proximal RA activation is earlier than the proximal CS activation. During PAC induced by the mapping catheter manipulation (arrow, the catheter was in high RA), the proximal RA activation is almost simultaneous to the distal RA activation and the distal CS atrial activation is earlier than the proximal CS atrial activation. **(E)** and **(F)** Radiographs of RA, CS, and ABL catheters. 3D, three dimensional; ECG, electrocardiogram; PAC, premature atrial contraction; SAN, sinoatrial node; ABL, ablation catheter; LAO, left anterior oblique projection; RAO, right anterior oblique projection.

**Figure 2:**
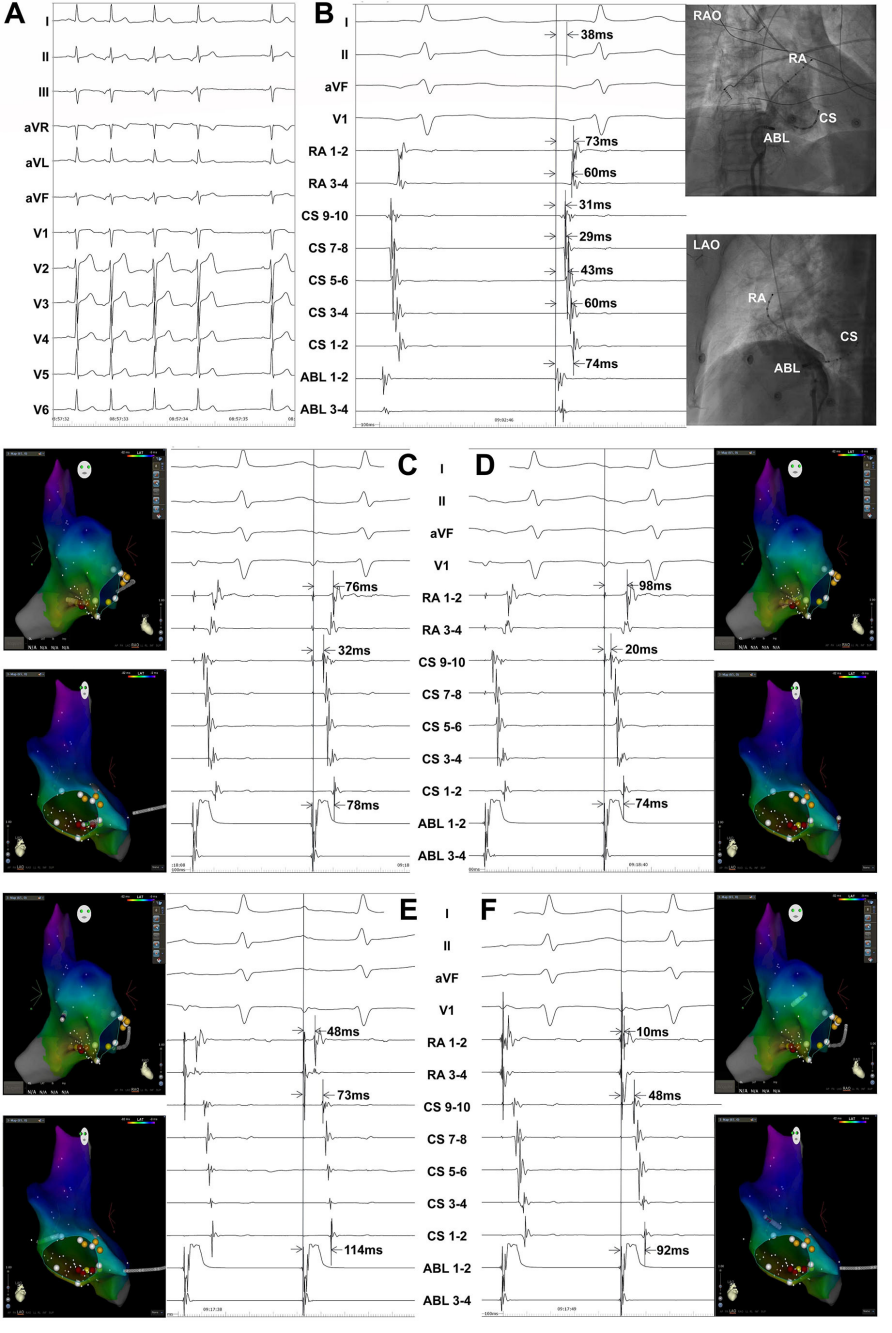
Atrial pacing at different sites in a patient with PACs originating from inferior vena cava showing the atrial activation sequences and activation times of the right atrium (RA) and coronary sinus (CS). Surface ECG leads I, II, aVF, V_1_, RA_1−2_~RA_3−4_, CS_9−10_~CS_1−2_, and ABL_1−2_~ABL_3−4_ were simultaneously recorded. The RA catheter was advanced in the right atrial appendage. **(A)** Twelve-lead surface ECG monitoring at baseline. A negative P wave was seen in the inferior leads and the precordial leads. A positive P wave was seen in leads I, aVR, and aVL. **(B)** The ablation catheter was located at the successful ablation target site. ABL_1−2_-P interval = 38 ms, ABL_1−2_-CS_1−2_ interval = 74 ms, ABL_1−2_-CS_3−4_ interval = 60 ms, ABL_1−2_-CS_5−6_ interval = 43 ms, ABL_1−2_-CS_7−8_ interval = 29 ms, ABL_1−2_-CS_9−10_ interval = 31 ms, ABL_1−2_-RA_1−2_ interval = 73 ms, and ABL_1−2_-RA_3−4_ interval = 60 ms during a spontaneous PAC. **(C)** ABL_1−2_-CS_1−2_ interval = 78 ms, ABL_1−2_-CS_9−10_ interval = 32 ms, and ABL_1−2_-RA_1−2_ interval = 76 ms when pacing near the ablation target site by the ablation catheter. **(D)** ABL_1−2_-CS_1−2_ interval = 74 ms, ABL_1−2_-CS_9−10_ interval = 20 ms, and ABL_1−2_-RA_1−2_ interval = 98 ms when pacing at CS ostium by the ablation catheter. **(E)** ABL_1−2_-CS_1−2_ interval = 114 ms, ABL_1−2_-CS_9−10_ interval = 73 ms, and ABL_1−2_-RA_1−2_ interval = 48 ms when pacing at the right atrial free wall by the ablation catheter. **(F)** ABL_1−2_-CS_1−2_ interval = 92 ms, ABL_1−2_-CS_9−10_ interval = 48 ms, and ABL_1−2_-RA_1−2_ interval = 10 ms when pacing at the basal right atrial appendage by the ablation catheter. ECG, electrocardiogram; PAC, premature atrial contraction; ABL, ablation catheter; LAO, left anterior oblique projection; RAO, right anterior oblique projection.

The atrial activation sequences of RA and CS, as well as the relative timing between the RA and CS catheter were used to distinguish spontaneous PACs from PACs induced by catheter manipulation during the mapping procedure (Figure 1). The baseline atrial activation sequences and atrial activation times of RA and CS were recorded. During the mapping procedure, changes in atrial activation sequence or atrial activation time during a PAC indicate that the PAC was provoked by catheter movement. The feasibility of this mapping method was also proved by comparing the atrial activation sequences and atrial activation times of RA and CS during the pacing of the atrium at different sites (Figure 2). A similar atrial activation sequence and activation time of RA and CS, respectively, were observed during pacing near the site of origin of the PAC. However, a significantly different atrial activation sequence and/or atrial activation time of RA and CS, respectively, were produced by pacing the atrium far from the origin site of PAC.

For PACs with a left-side origin, transseptal procedures with long Swartz sheaths (L1-type, St. Jude Medical, Minneapolis, MN) were performed to map the left atrium. A bolus dose of 100 IU/kg of heparin was introduced for anticoagulation once the sheaths entered the left atrium. The activated clotting time (ACT) was maintained at a range of 300–350 s. Three-dimensional (3D) electroanatomic mapping was performed using the CARTO 3 system (Biosense Webster, Inc., Diamond Bar, CA).

### Mapping and Ablation

Contact force-sensing irrigated ablation catheters (ThermoCool SmartTouch, Biosense Webster, Inc., Diamond Bar, CA) were used for mapping and ablation. If necessary, a 20-pole PentaRay mapping catheter (Biosense Webster, Inc., Diamond Bar, CA) was also used for mapping. The earliest atrial activation during PACs was identified as the target ablation site. Radiofrequency energy was initially delivered at 30–35 W and 43°C for 10 s at the site which recorded the earliest atrial activation during PACs. If PACs disappeared within 10 s, additional energy was applied at the same site for 60 s. Consolidated ablation around the target site would be performed if necessary. If PACs did not disappear within 10 s, ablation was stopped and remapping was performed. An acute successful ablation of PACs was defined as the disappearance of PACs for at least 30 min after radiofrequency energy application, as well as burst atrial pacing or isoproterenol infusion could not induce PACs anymore.

For PACs arising from pulmonary veins, circumferential pulmonary vein isolation (CPVI) was performed to isolate the “culprit” ipsilateral pulmonary veins. Ablation index (AI) was adopted to guide ablation. Ablation energy was set at 35–40 W and 43°C. The AI was set at 380 in sites on the left atrium posterior wall and 450 in sites on the left atrium anterior wall, respectively.

For PACs arising from superior vena cava (SVC), SVC isolation would be performed. To avoid right phrenic nerve injury, the course of the phrenic nerve was identified by pacing method using the ablation catheter. Ablation energy was set at 30–35 W and 43°C. The AI was set at 380.

Fentanyl was used to relieve pain during ablation. After intravenous injection of a bolus dose of 0.05–0.1 mg, an additional maintenance dose of 0.05 mg/h fentanyl was administrated during the procedure. After CPVI or SVC isolation, a 30-min period with isoproterenol infusion would be taken to estimate whether pulmonary veins or SVC reconnected.

### Quality of Life Assessment

To assess the effect of RFCA on the quality of life, all patients completed the quality of life questionnaire SF-36 before the procedure and at 3 months after RFCA.

### Post-Ablation Follow Up

Anticoagulation therapy (Warfarin, Dabigatran, or Rivaroxaban) was applied in patients with PACs originating from the pulmonary veins. No patient received any ADDs (including calcium channel blockers and β-blockers) during follow-up. Surface ECGs and 24-h Holter monitoring were obtained prior to discharge. All patients were followed up in the outpatient clinic, and 24-h Holter monitoring was performed again during a 3-month follow-up. Freedom from the recurrence of PACs (the number of PACs was <100 per 24 h) during follow-up was defined as clinical success.

### Statistical Analysis

Continuous variables were expressed as mean ± standard deviation and were compared using Student's *t*-test. All statistical analyses were performed using SPSS 16.0 for Windows (SPSS Inc., Chicago, IL, USA). A *p* < 0.05 was considered statistically significant.

## Results

### Patient Characteristics

A total of 43 patients (23 men and 20 women) with an average age of 52.6 ± 17.6 years were finally enrolled in the study. The mean PACs number was 21,685 ± 9,596 per 24 h (range, 10,018–39,287) and the mean PACs burden was 28.9 ± 13.7% (range, 10.6–60.3%). The patient characteristics are summarized in Table 1.

**Table 1:** Patient characteristics.

**Variables**	**All (*n* = 43)**
Male, *n* (%)	23 (53.5)
Age, y	52.6 ± 17.6
BMI, Kg/m^2^	24.8 ± 2.8
History of PACs, mon	8.1 ± 12.8
No. of PACs per 24 h	21,685 ± 9,596
Mean PACs burden (%)	28.9 ± 13.7
**Symptoms**	
Palpitation, *n* (%)	37 (86)
Chest tightness, *n* (%)	6 (14)
Breathless, *n* (%)	4 (9.3)
**Comorbidity**	
Hypertension, *n* (%)	16 (37.2)
Diabetes, *n* (%)	3 (7)
Ischemic stroke, *n* (%)	5 (11.6)
Chronic kidny disease, *n* (%)	3 (7)
Hyperlipoidemia, *n* (%)	5 (11.6)
Premature ventricular contraction, *n* (%)	5 (11.6)
Total cholesterol, mmol/L	3.95 ± 0.83
LDL-C, mmol/L	2.48 ± 0.83
LAD, mm	33.7 ± 4.8
LVEDD, mm	45 ± 3.6
LVEF,%	64.9 ± 6.3

*PAC, premature atrial contraction; BMI, body mass index; LDL-C, low density lipoprotein cholesterol; LAD, left atrium diameter; LVEDD, Left ventricular end diastolic diameter; LVEF, left ventricular ejection fraction*.

### Mapping and Ablation

All the patients underwent successful electrophysiological mapping and ablation without any complications. Spontaneous clinical PACs were observed in 37 (86%) patients. In the other 6 patients, isoproterenol infusion was performed to induce PACs. Short runs of atrial tachycardia (<5 atrial beats) were recorded in 32 patients (74.4%). The most common target sites were pulmonary veins, CS ostium, and crista terminalis (Table 2).

**Table 2:** Parameters of mapping and ablation.

**Variables**	**All (*n* = 43)**
**RFCA procedural parameters**	
Procedure time (min)	108 ± 24.8
Ablation time (sec)^*^	96 ± 16
Number of Ablation^*^	4 ± 2
**Pulmonary veins isolation**	
LSPV isolation time (min)	41 ± 6.7 (*n* = 6)
RSPV isolation time (min)	31 ± 5.7 (*n* = 6)
**Location of Ablation target site**	
Superior vena cava, *n*	3
Inferior vena cava, *n*	2
High crista terminalis, *n*	3
Low crista terminalis, *n*	3
Coronary sinus ostium, *n*	7
Tricuspid annulus, *n*	2
Right atrial septum, *n*	2
Left atrial septum, *n*	2
Left superior pulmonary vein, *n*	6
Right superior pulmonary vein, *n*	6
Mitral annulus, *n*	2
Left atrial roof, *n*	1
Left atrial posterior wall, *n*	1
Left atrial appendage, *n*	1
Right coronary cusp, *n*	1
Non coronary cusp, *n*	1
Local A-P interval, (ms)	36 ± 7.6
Concomitant PACs run^#^, *n* (%)	32 (74.4)

* *Not including pulmonary veins isolation and superior vena cava isolation*.

# *Indicating short runs with <5 atrial beats*.

**Figure 3:**
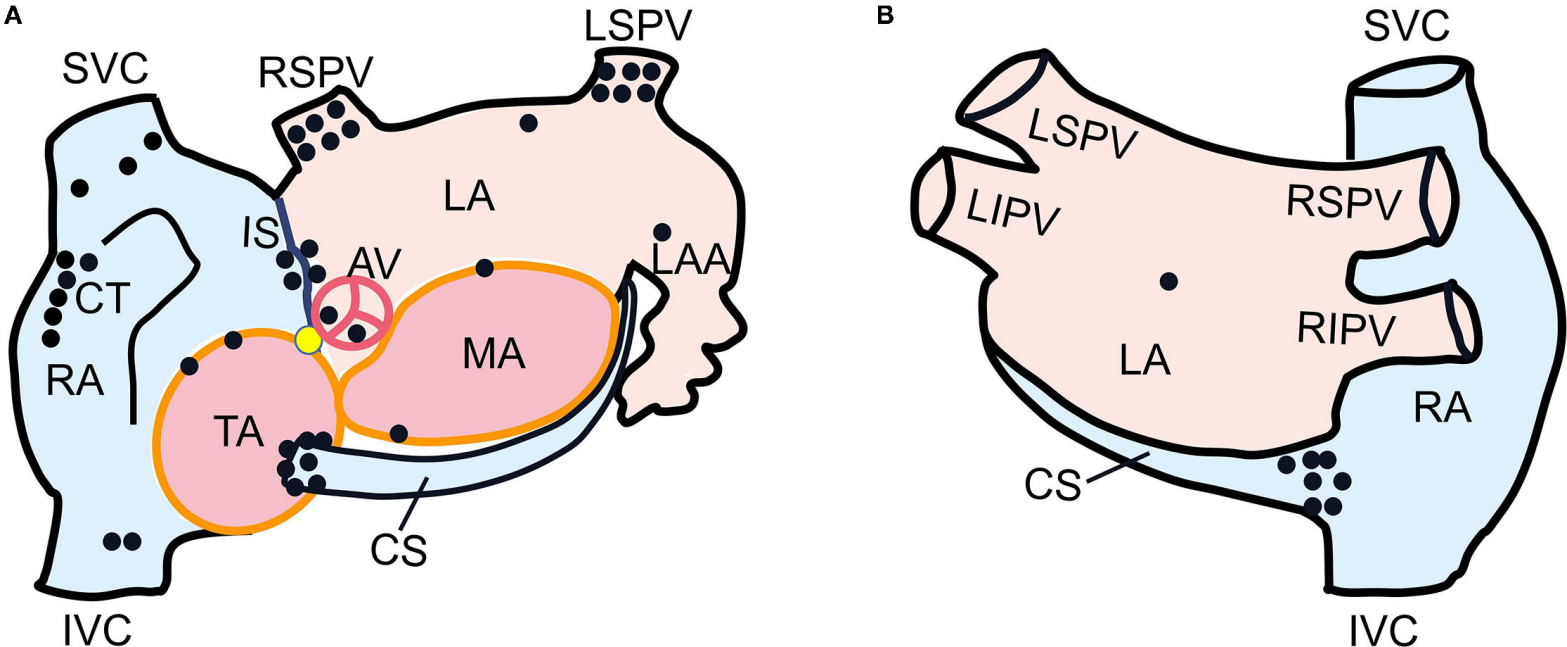
Schematic diagram of left anterior oblique **(A)** and postero-anterior **(B)** views of dual atria showing the successful ablation target sites in 43 patients. Each black dot represented one ectopic focus. The yellow dot indicated His bundle. SVC, superior vena cava; IVC, inferior vena cava; RA, right atrium; CT, crista terminalis; CS, coronary sinus; TA, tricuspid annulus; IS, interatrial septum; LSPV, left superior pulmonary vein; RSPV, right superior pulmonary vein; MA, mitral annulus; LA, left atrium; LAA, left atrial appendage; AV, aortic valve.

Figure 3 shows the successful ablation target sites in 43 patients, with the locations as follows: superior vena cava (*n* = 3), inferior vena cava (*n* = 2), interatrial septum (*n* = 4), crista terminalis (*n* = 6), CS ostium (*n* = 7), tricuspid annulus (*n* = 2), left superior pulmonary vein (*n* = 6), right superior pulmonary vein (*n* = 6), mitral annulus (*n* = 2), left atrium appendage (*n* = 1), left atrium posterior wall (*n* = 1), left atrial roof (*n* = 1), non-coronary cusp (*n* = 1), and right coronary cusp (*n* = 1).

**Figure 4:**
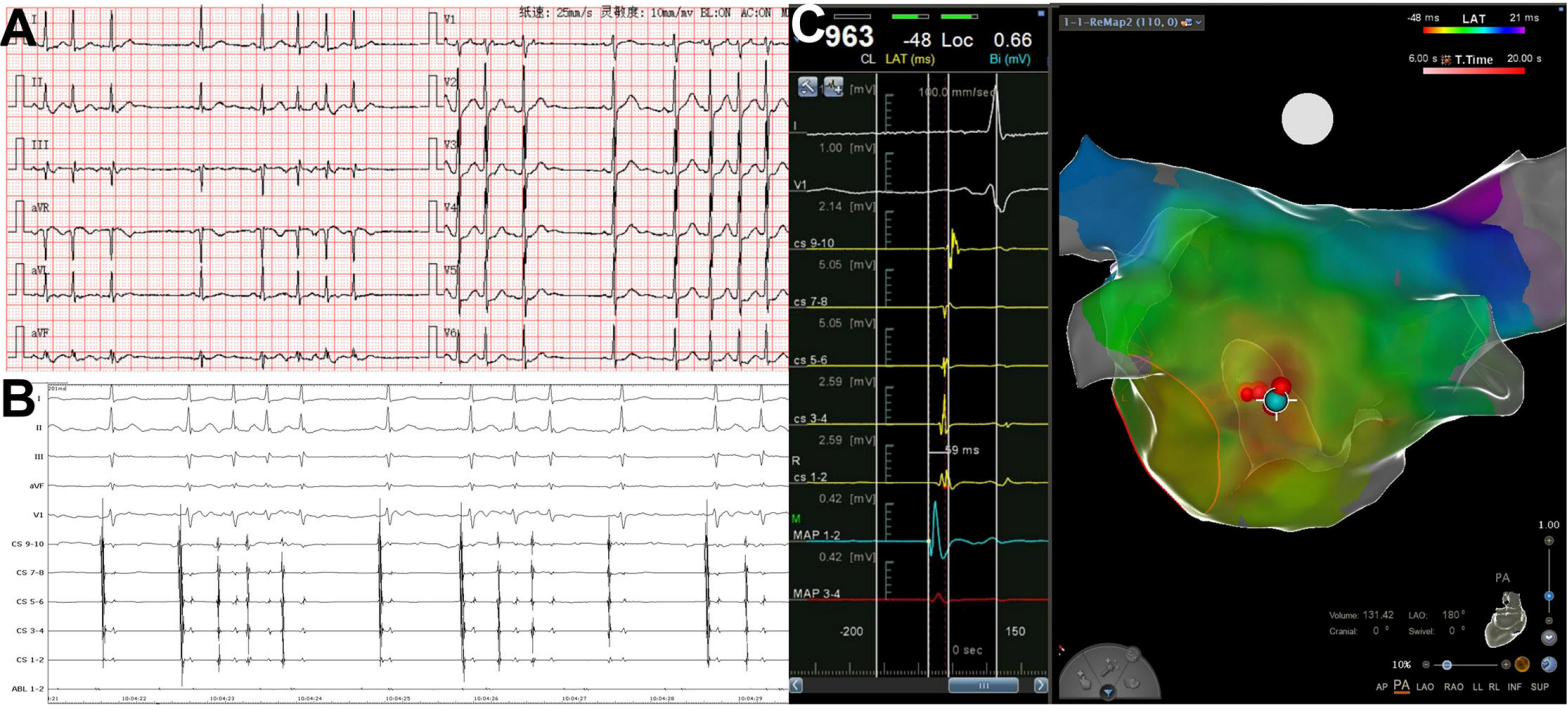
PACs with short runs arising from left atrial posterior wall. **(A)** Twelve-lead surface ECG tracing during PACs. The P wave morphology is positive in precordial leads and inferior leads, flat in leads I and aVL, and negative in lead aVR. **(B)** Simultaneous recording of surface ECG lead I, II, III, aVF, V_1_, CS_9−10_~CS_1−2_, and ABL_1−2_. The atrial activation in CS electrodes is almost at the same time. **(C)** The 3D mapping shows the origination of PACs in posterior–anterior view. The blue dot indicates the earliest activation point of PACs, and red dots indicate the ablation points. The atrial activation in the blue dot precedes surface ECG P wave in lead V_1_ by 59 ms. 3D, threedimensional; CS, coronary sinus; ECG, electrocardiogram; PAC, premature atrial contraction; ABL, ablation catheter.

The activation time at the successful ablation target sites preceded the surface ECG P-wave onset by 36 ± 7.6 ms (range, 25–59 ms, Figure 4). Six patients with left superior pulmonary vein origin underwent left pulmonary vein isolation. Six patients with right superior pulmonary vein origin underwent right pulmonary vein isolation. SVC isolation was performed in 3 patients.

**Figure 5:**
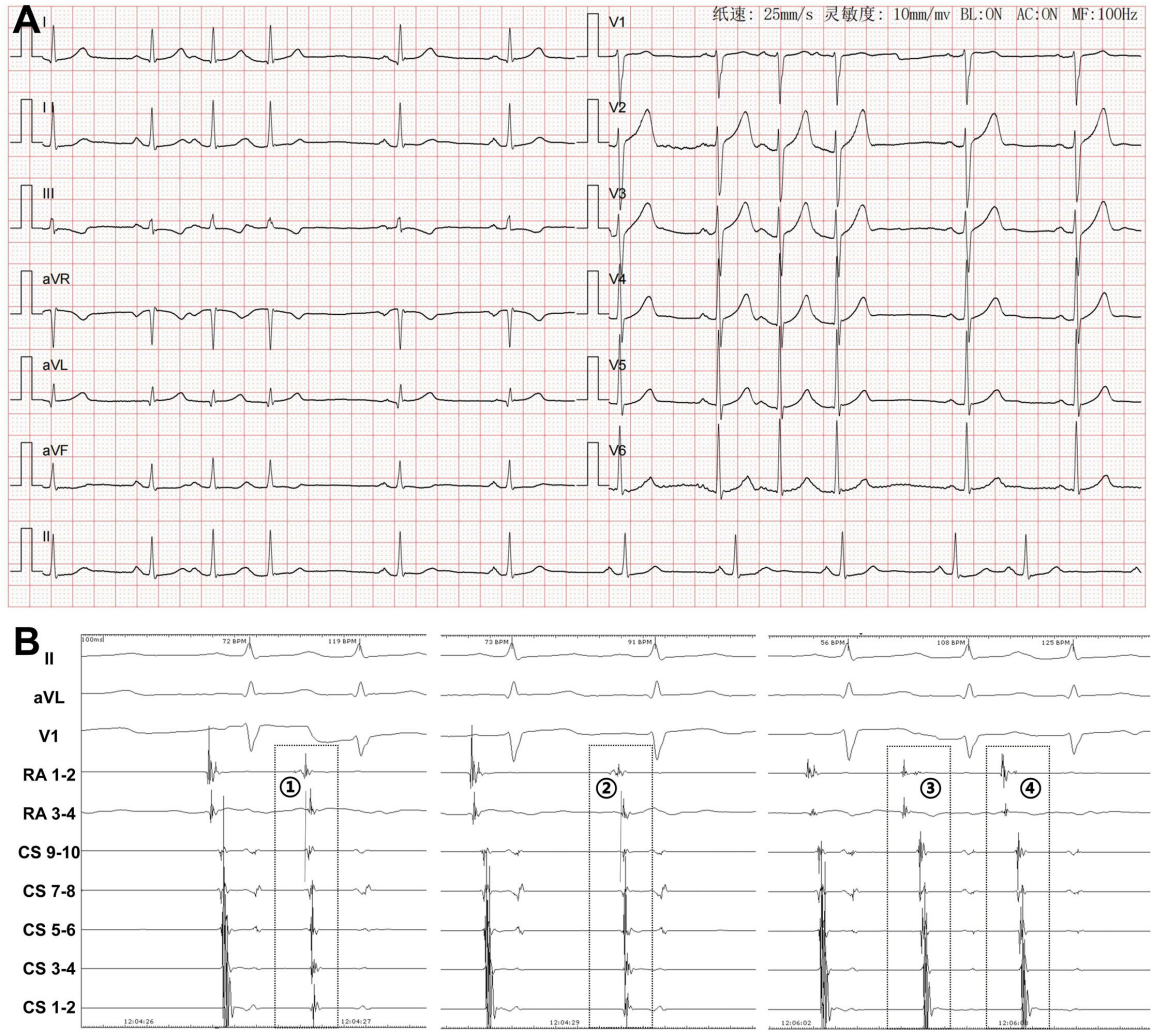
Differentiation of spontaneous PACs and PACs induced by catheter manipulation during mapping procedure. **(A)** Twelve-lead surface ECG tracing during PACs. The P wave morphology is positive in precordial leads, inferior leads, and lead I, and negative in lead aVR and isoelectrical in lead aVL. **(B)** Simultaneous recording of surface ECG leads II, aVL, V_1_, RA_1−2_, RA_3−4_, and CS_9−10_~CS_1−2_. During spontaneous PAC (PAC), the distal RA activation is earlier than the proximal RA activation and the atrial activation sequence in CS is from proximal to distal. The atrial activation in CS_9−10_ is earlier than that in RA_3−4_. During PAC induced by the catheter manipulation (PAC~), both the RA and the CS atrial activation time and sequences are useful for differentiation. At first glance, PAC may be thought to be a spontaneous PAC. However, different coupling intervals, as well as the fact that the atrial activation in RA_3−4_ is earlier than that in CS_9−10_ confirm it as a manipulation-induced one. In PAC , the atrial activation in RA_3−4_ is earlier than that in RA_1−2_. In PAC , the atrial activation in RA_3−4_ is significantly earlier than that in CS_9−10_. These differences indicate that both PAC and PAC are induced by catheter manipulation. RA, right atrium; CS, coronary sinus; ECG, electrocardiogram; PAC, premature atrial contraction.

During the mapping procedure, the atria activation sequences, as well as the relative timing between the RA and CS were used to distinguish spontaneous PACs from PACs induced by catheter manipulation (Figure 5). PACs can be easily distinguished using this method.

### Clinical Effects

Twelve patients who underwent pulmonary vein isolation took dabigatran (*n* = 6) or rivaroxaban (*n* = 6) for anticoagulation for 2 months. During the 15 ± 8 months of follow-up, the recurrence of PACs was observed only in 2 patients. Besides, the number of PACs per 24 h in the recurrent 2 patients was significantly reduced compared with the baseline (8,685 vs. 19,810 and 7,436 vs. 18,910, respectively). The mean PACs number (396 per 24 h) and the mean PACs burden (0.5%) at 3 months after RFCA were significantly reduced compared to baseline (*p* < 0.05 for both). The quality of life, estimated by the quality of life questionnaire, was also markedly improved at 3 months after the RFCA procedure compared with the baseline (Table 3).

**Table 3:** Quality of life scores in pre- and postablation.

	**PF**	**RP**	**BP**	**GH**	**VT**	**SF**	**RE**	**MH**
Preablation	75 ± 19	29 ± 33	63 ± 19	36 ± 17	59 ± 15	54 ± 22	35 ± 32	69 ± 21
3 Months postablation	94 ± 23^*^	65 ± 36^*^	77 ± 14^*^	62 ± 10^*^	70 ± 12^*^	73 ± 19^*^	78 ± 35^*^	85 ± 15^*^

* *P < 0.05 when compared to preablation*.

*PF, physical functioning; RP, role-physical; BP, bodily pain; GH, general health; VT, vitality; SF, social functioning; RE, role-emotional; MH, mental health*.

## Discussion

### Main Findings

The major findings in the present study were that: (1) RFCA was feasible, safe, and effective to eliminate isolated frequent, symptomatic, and drug-refractory PACs in patients with structurally normal heart; (2) The patients' quality of life was significantly improved after ablation.

### Mapping and Ablation of PACs

Similar to the localization of premature ventricular contraction, the origin of PACs can approximately be located by the morphology of the P wave in surface ECG (11, 12). However, PACs are usually merged with the T wave of the previous beat, making it hard to distinguish the morphology of the P wave. More importantly, during the mapping procedure, catheter manipulation can provoke PACs, which also complicate mapping due to the small amplitude of the P wave. Pace mapping is therefore challenging to differentiate P wave caused by PACs from that generated by pacing. All the above factors would make the mapping of PACs more challenging. Previous studies (13, 14) have shown that the interatrial and intraatrial conduction time differences, as well as atrial activation sequences were useful to distinguish the origins of focal AT. In our study, we applied a quadripolar electrode catheter to the HRA or RAA for recording and mapping combined with the CS catheter. The activation sequences of RA and CS, as well as the atrial activation times from proximal to distal RA, from proximal to distal CS, and from RA to CS were useful to distinguish spontaneous PACs from PACs induced by catheter manipulation (Figures 1, 5). Furthermore, in addition to the comparison of atrial activation sequences in multiple catheters, reproducibility of the PACs with the same coupling interval during a procedure is also helpful to distinguish clinical and catheter-induced PACs.

### Rationale and Indications of PACs Ablation

Catheter ablation of premature ventricular contraction has been a common procedure in the electrophysiology lab. However, less attention is paid to the ablation of PACs. It is probably because they seem to be more benign than premature ventricular contraction. But actually, patients with frequent PACs, especially with short runs of atrial tachycardia (<5 beats), usually presented with obvious symptoms, significantly affecting their quality of life. Also, the burden of PACs has been reported to be associated with an increased risk of atrial fibrillation, supraventricular tachycardia, and stroke (15–18). Furthermore, both frequent PACs and PACs run are independent predictors of late recurrence of atrial fibrillation ablation (10). Among them, catheter ablation of PACs (especially pulmonary veins origin) can increase the success rate of atrial fibrillation ablation (4, 19). Moreover, frequent PACs may cause cardiomyopathy (7–9), which can also be reversed by RFCA.

Although no guideline recommendations or expert consensus on RFCA for PACs are available to date, an increasing body of evidence demonstrates the feasibility and efficacy of RFCA for eliminating PACs. Our findings indicate that symptomatic, frequent, and drug-refractory PACs, with or without concomitant atrial fibrillation, could be recommended for ablation. In the present study, all patients were excluded for the existence of structural heart diseases and atrial fibrillation. The application of PACs ablation could significantly improve their quality of life, suggesting the symptom is one of the main considerations. In our study, part of the patients received RFCA due to refusal to accept AADs.

### Study Limitations

One limitation is that the present study is an observational study. Empirically, an additional RA catheter could be useful to distinguish spontaneous PACs from PACs induced by catheter manipulation, facilitating the mapping procedure. However, we did not compare the mapping time and the efficacy of catheter ablation between patients undergoing catheter ablation with or without using the RA catheter. Further studies on this issue are warranted in the future. Secondly, as our study excluded patients with atrial tachycardia (≥5 beats), atrial flutter, or atrial fibrillation, it remains unclear if the ablation of PACs can prevent sustained atrial tachycardia or atrial fibrillation, although the quality of life was markedly improved 3 months after RFCA. A prospective randomized controlled trial would be helpful to answer this question. Thirdly, the sample size in the present study is limited, and it would overestimate the success rate of ablation to some extent. Also, the limited sample size may explain the different distribution of PACs' location sites when compared with previous studies (4, 5). Fourthly, the infusion of isoproterenol was used to induce PACs before ablation and evaluate the effect after ablation in our study. However, isoproterenol infusion of can also unmask underlying atrial tachycardia or trigger atrial fibrillation (clinical or non-clinical), which may affect the patient's enrollment and exclusion.

## Conclusions

Radiofrequency catheter ablation was feasible, safe, and effective to eliminate isolated frequent, symptomatic, and drug-refractory PACs in patients with a structurally normal heart. The activation sequences, as well as the atrial activation times in multiple catheters were useful to distinguish spontaneous PACs from catheter-induced PACs during the mapping procedure. The elimination of PACs by RFCA significantly improved symptoms and the quality of life.

## Data Availability Statement

The raw data supporting the conclusions of this article will be made available by the authors, without undue reservation.

## Ethics Statement

The studies involving human participants were reviewed and approved by Zhongnan Hospital of Wuhan University. The patients/participants provided their written informed consent to participate in this study. Written informed consent was obtained from the individual(s) for the publication of any potentially identifiable images or data included in this article.

## Author Contributions

ZL conceived and planned this trial. BH wrote the manuscript with support from YL. WH and SY carried out a part of this trial. WY performed the computations. FZ and XW verified the analytical methods. SP revised the whole manuscript. All authors discussed the results and contributed to the final manuscript.

## Funding

This work was supported by Grants from the National Natural Science Foundation of China (82070425 to ZL), Natural Science Foundation of Hubei Province of China (2021CFA011 to ZL), Health Commission of Hubei Province Scientific Research Project (WJ2021Q039 to BH), Translational Medicine and Interdiscipline Research Joint Fund of Zhongnan Hospital of Wuhan University (ZNLH201907 to ZL), and Science and Technology Innovation Cultivation Fund of Zhongnan Hospital of Wuhan University (ZNPY2019005 to ZL).

## Conflict of Interest

The authors declare that the research was conducted in the absence of any commercial or financial relationships that could be construed as a potential conflict of interest.

## Publisher's Note

All claims expressed in this article are solely those of the authors and do not necessarily represent those of their affiliated organizations, or those of the publisher, the editors and the reviewers. Any product that may be evaluated in this article, or claim that may be made by its manufacturer, is not guaranteed or endorsed by the publisher.
